# The Effect of Exercise upon the Teeth

**Published:** 1895-01

**Authors:** A. Hugh Hipple

**Affiliations:** Omaha.


ARTICLE V.

### THE EFFECT OF EXERCISE UPON THE TEETH.

BY A. HUGH HIPPLE, L. D. S., D. D. S., OMAHA.

The action of the American Medical Association in establishing a dental section, and thereby recognizing dentistry as a specialty of medicine, has not only conferred dignity upon that calling and secured for its practitioners recognition as professional men, but it has also furnished them with an incentive to familiarize themselves with those broad principles that underlie the practice of medicine. On the other hand it has called the attention of physicians to the fact that the condition of the teeth has much to do with the condition of the general system, and patients are nowadays referred by them to the dentist quite as frequently as to the surgeon. But although physicians are beginning to recognize the seriousness of diseases of the teeth, and to impress upon their patients the importance of having them properly attended to whenever they show signs of decay, the fact that a rapid deterioration of these organs is now going on and that serious results are likely to follow this deterioration has been almost entirely overlooked. A little inquiry and investigation, however, must convince anyone that such a change for the worse is taking place. Children rarely have as good teeth as their parents had at the same age, but on the contrary their teeth are often almost hopelessly decayed before the dentures of their parents show signs of impairment. A century ago the city of New York, with a population of about fifty thousand, had only one dentist, and although much less attention was paid to the teeth then than is paid to them now, there is abundant evidence to show that our forefathers had better teeth than we have. Dr. Hammond tells us that the coming man will be hairless and toothless, and the tendency certainly seems to be in that direction. The conservation of the natural teeth is the end toward which every intelligent and conscientious dentist directs his efforts, but if the quality of the teeth continues to deteriorate the replacement of lost dental tissue must gradually give way to the replacement of lost dental organs. Every physician knows, of course, that this would not be conducive to health, but that medical men generally do not realize how important a bearing the condition of the teeth has upon the health and longevity of an individual is shown by the fact that in examining applicants for life insurance no question regarding the condition of the mouth is ever asked. The teeth may be so badly decayed that the proper mastication of food is impossible, the saliva may be vitiated, the gums and alveoli may be the seat of abscesses that are continually discharging pus, but these conditions do not concern the examining physician. Probably not one in a thousand, in reporting upon the application of a man twenty-five years of age, would think of mentioning the fact that he had lost, say, all his upper teeth and half his lower ones, although after giving it a little thought few would probably care to dispute the statement that the loss of the teeth before the twenty-fifth year will on the average shorten the life of an individual by at least several years. If this be true, no apology is needed for calling the attention of medical men to the dental deterioration that has been referred to, for discussing its propable causes, and for suggesting possible remedies.

In endeavoring to ascertain why it is that imperfect dentures are so common and dental diseases so alarmingly prevalent, it must be born in mind that although the teeth are vital organs, developed and nourished very much like the other organs of the body, they differ from them widely in susceptibility to disease. While other organs may be delicate early in life and afterward become quite strong and healthy, or *vice versa*, the teeth, if not impaired by disease, remain the same, with the exception, perhaps, of a slight increase of density as age advances. All the other organs of the body, too, including the bones, are endowed with recuperative powers whereby injuries are to a greater or less extent repaired; but the teeth possess no such attributes, and are apparently governed by somewhat different laws from those that regulate other parts of the animal economy. Teeth that are perfect in form and structure are rarely, if ever, attacked by decay. It is only where the enamel is defective that dental caries can obtain a foothold. As the teeth are developed early in life, it is during childhood that those influences are exerted which by interfering with their development predispose them to disease, and it is to childhood, therefore, that we must look for the causes of dental deterioration.

Scientists tell us that use and disuse have much to do with the development of organs, and that with the progress of civilization the brain has increased and the jaws have decreased in size. The wisdom teeth from disuse have degenerated and become rudimentary; the canines, being no longer needed to tear flesh from the bone and do other heavy work, have become smaller and less prominent; the teeth in general have become soft and chalky and very susceptible to decay. This, they say, is the result of the substitution of soft, well-cooked food for that which required vigorous use of the teeth and masticatory muscles, and as there seems to be no liklihood of civilized man going back to primeval methods of preparing food, the inference is that dental deterioration is one of the prices we are obliged to pay for a high state of civilization. But use and disuse not only modify the size and structure of organs when persisted in for a series of generations, but their effect upon the organs of any one individual are no less marked. Tie up an arm so that it cannot be used, and the muscles will soon become soft and flabby, and will eventually disappear. Lock up a child in a room by itself with nothing to occupy its thoughts, and it will in time become an imbecile. It appears that a certain amount of exercise is essential to the development of most organs. A part when performing work requires and receives more blood than when at rest, and if much work is performed the blood vessels increase in size and the part is better nourished. That a close relationship exists between development and nourisment, and between nourishment and exercise, is a fact so well known that it need not be discussed here; but so far as the study of the teeth is concerned the principle has been applied to the race rather than to the individual. It is undoubtedly true that what the people of a country eat for eight or ten generations will determine in a general way the size and shape of their jaws and the form and structure of their teeth at the end of that time; but it is probably no less true that what a child eats up to the time he is eight or ten years of age will determine just as certainly what will be the condition of his dental organs for the rest of his life. If the food of the child is such as requires vigorous use of the jaws, the blood supply will be liberal, the parts will be well developed, and the teeth will not be likely to suffer from decay. On the other hand, if the child is fed on soft food, requiring little or no active mastication, the jaws and teeth will be poorly nourished, and the latter at least will be defective in structure. Erupted into the mouth in that condition, no amount of care can protect them from the ravages of decay, which will sooner or later impair their usefulness and mar their beauty.

It must be remembered in this connection that although none of the temporary teeth make their appearance in the mouth until the child is five or six months of age, their crowns are almost fully developed at birth, and that the jaws of a newly born child also contain the germs of twenty-four of the permanent teeth in various stages of development. These permanent teeth do not begin to erupt until the child is about six years of age, but during that time the process of calcification is continually going on. With the first molars, the incisors and the canines, it is well started by the end of the first year; with the bicuspids, at the end of the second year; and with the second molars at about the fifth year. It will thus be seen that between the second and fifth years this process of calcification upon which depends the future character of the teeth is in most active progress. Nature intended that during this period the jaws and teeth should be well exercised, and to that end provided the child with a perfect temporary set of teeth, but, as a matter of fact, they are used but very little compared with the other organs of the body The muscles of the arms, legs and head are in almost constant use, and are consequently always well supplied with blood. The brain is wrestling with the problems of life as they present themselves, and it, too, is being exercised and developed. The eye is being trained to examine every object, and the ear to catch the slightest sound, but the teeth are hardly used at all. Nine out of ten mothers feed their children of that age on soft food. Bread made from fine flour, biscuits soaked in tea or milk, meat cooked tender and cut into small bits, with potatoes and other vegetables in such a condition that they require little or no mastication, form the chief food of the little three-year-olds. Not being actively exercised, the teeth and jaws need but a small quantity of blood, and, owing to the imperfect development that results from insufficient nourishment, they are unable to resist the attacks of the pathogenic germs that are always present in the mouth, and which eventually destroy them.

The remedy is in the hands of parents. If they will see that their children, at the earliest possible age, use their first teeth vigorously, they need have little anxiety in regard to the second set. In other words, if a demand is created for sound, solid teeth, nature will be almost certain to supply them. It is by no means difficult to teach children to chew their food. Nothing pleases small children more than to be allowed to nibble a hard biscuit or bite the meat from a bone. Nature prompts them to exercise their teeth in that way, just as it prompts a puppy to spend hours gnawing at a bone which has long since been stripped of its meat. But the average mother, partly, no doubt, out of respect to dainty dresses and well-kept carpets, but more particularly from fear of possible injury to the teeth themselves, objects to the dental calisthenics in which the child would gladly indulge, and thereby unconsciously opposes the efforts of nature to develop good teeth. Since teeth that are perfect in structure rarely if ever decay, an ounce of prevention in the way of developing healthy dental organs is certainly worth more than a pound of cure after they are diseased; and if parents will supply their children with an abundance of bone-producing food, see that the teeth are kept clean, have them examined and attended to from time to time by a competent dentist, and above all, have them well exercised by chewing hard food, nature will do her part, and their children in after years will rejoice in the possession of that almost priceless endowment, a beautiful and complete set of teeth.—*Dominion Dental Journal.*

